# Dissecting the Function and Assembly of Acentriolar Microtubule Organizing Centers in *Drosophila* Cells In Vivo

**DOI:** 10.1371/journal.pgen.1005261

**Published:** 2015-05-28

**Authors:** Janina Baumbach, Zsofia Anna Novak, Jordan W. Raff, Alan Wainman

**Affiliations:** Sir William Dunn School of Pathology, University of Oxford, Oxford, United Kingdom; Stowers Institute for Medical Research, UNITED STATES

## Abstract

Acentriolar microtubule organizing centers (aMTOCs) are formed during meiosis and mitosis in several cell types, but their function and assembly mechanism is unclear. Importantly, aMTOCs can be overactive in cancer cells, enhancing multipolar spindle formation, merotelic kinetochore attachment and aneuploidy. Here we show that aMTOCs can form in acentriolar *Drosophila* somatic cells in vivo via an assembly pathway that depends on Asl, Cnn and, to a lesser extent, Spd-2—the same proteins that appear to drive mitotic centrosome assembly in flies. This finding enabled us to ablate aMTOC formation in acentriolar cells, and so perform a detailed genetic analysis of the contribution of aMTOCs to acentriolar mitotic spindle formation. Here we show that although aMTOCs can nucleate microtubules, they do not detectably increase the efficiency of acentriolar spindle assembly in somatic fly cells. We find that they are required, however, for robust microtubule array assembly in cells without centrioles that also lack microtubule nucleation from around the chromatin. Importantly, aMTOCs are also essential for dynein-dependent acentriolar spindle pole focusing and for robust cell proliferation in the absence of centrioles and HSET/Ncd (a kinesin essential for acentriolar spindle pole focusing in many systems). We propose an updated model for acentriolar spindle pole coalescence by the molecular motors Ncd/HSET and dynein in conjunction with aMTOCs.

## Author Summary

During cell division, chromosomes are divided into two daughter cells by the mitotic spindle, a complex structure made from microtubules. The correct formation of the mitotic spindle is essential, as missegregation of chromosomes can lead to cell death or cancer. Therefore several mechanisms cooperate in nucleating the microtubules needed for the mitotic spindle and focusing them into a bipolar structure. One of these mechanisms, which has only recently been identified, is microtubule nucleation by acentriolar microtubule organizing centers (aMTOCs). These structures have been observed in several cell types, notably also in cancer cells, but is not known how they are formed and which function they might have in mitotic spindle assembly. We identified the pathway of aMTOC formation in *Drosophila*, which enabled us to perturb their formation in order to study their role during spindle formation. We show that aMTOCs are a source of microtubule nucleation, but their contribution to spindle formation is normally masked by other, more dominant, pathways of microtubule nucleation. Furthermore, we have identified a role for aMTOCs in focusing of mitotic spindle poles by the molecular motor dynein in cells in which centrioles are missing.

## Introduction

Centrosomes are the major microtubule organizing centers (MTOCs) in many cells, and they consist of a pair of centrioles surrounded by a cloud of pericentriolar material (PCM), which contains proteins that nucleate and stabilize microtubules (MTs). For many years centrosomes were thought to be the sole drivers of mitotic spindle assembly in animal somatic cells by nucleating MTs, which then randomly search and capture kinetochores in the cytoplasm to form a bipolar spindle [1]. More recently it has become clear that centrosomal MT nucleation cooperates with at least two other pathways of MT nucleation—the chromatin and augmin-mediated pathways—to drive efficient mitotic spindle assembly [2–4]. In current models, MT assembly is induced around the chromatin and the plus ends of these MTs are then captured by the kinetochores and continue to grow from there in a MT bundle [5–8]. This leads to the formation of kinetochore fibers (K fibers) with minus ends that are pushed away from the kinetochores. These K fibers then coalesce into a bipolar spindle together with astral MTs emanating from the centrosomes in a mechanism involving three distinct steps: First, K fibers become crosslinked by the kinesin-14 Ncd/HSET. Second, K fibers are captured by the tips of growing centrosomal MTs, which is also mediated by Ncd/HSET. Third, K fibers are then transported towards centrosomes along astral microtubules by dynein motors, leading to their integration into the bipolar spindle [6,9]. Furthermore, the augmin complex is required for spindle MT amplification by nucleating MTs that branch off the sides of existing MTs, which increases the density of MTs within the mitotic spindle [10–15].

While all three MT nucleation pathways normally make important contributions to spindle assembly, they appear to be partially redundant. Many cell types that usually have centrosomes can assemble bipolar mitotic spindles in their absence, solely relying on chromatin- and augmin-mediated MT nucleation—although spindle assembly without centrosomes takes longer and is usually less accurate [16–20]. Molecular motors are sufficient for focusing MTs into a bipolar spindle without the need for centrosomes to dictate the organization of the two spindle poles (we define spindle poles as the focused collection of MT minus ends of the spindle [21]). In this case the kinesin-14 Ncd/HSET becomes crucial as it crosslinks MTs in a centrosome-independent way; loss of kinesin-14 proteins has been shown to cause severe pole focussing defects in the absence of centrosomes in female meiosis in *Drosophila* and mouse oocytes, mitosis in *Arabidopsis*, and in *Xenopus* egg extracts [22–25]. The dynein complex also plays a crucial role in acentriolar spindle pole focusing in some systems such as acentriolar spindles reconstituted from *Xenopus* egg extract or from cell free extracts prepared from HeLa cells [25–28]. The exact mechanism by which dynein contributes to acentriolar pole focusing however is unclear, as its normal function in pole focusing relies on the transport of K fibers towards centrosomes [9], which are not present in this case.

While the chromatin-mediated and augmin-dependent MT nucleation pathways are well studied, our knowledge of other acentriolar mechanisms of MT nucleation during mitosis is limited. One such mechanism has been described in centrosome-free mouse oocytes and early mouse embryos where centrosome function is replaced by multiple acentriolar MTOCs (aMTOCs) to which the centrosomal proteins γ-tubulin and Pericentrin localise [29–31]. These aMTOCs form de novo in prophase in the cytoplasm and around the nuclear envelope, and a bipolar spindle is formed in later stages of meiosis through the progressive clustering of multiple aMTOCs into just two poles [30]. In contrast, much less is known about the nature and function of aMTOCs in somatic cells. The presence of γ-tubulin enriched aMTOCs that mediate the de novo formation of MTs has been described in acentriolar *Drosophila* cultured cells [32,33]. In acentriolar DT40 chicken cells, aMTOCs containing the pericentriolar proteins CDK5RAP2 and γ-tubulin that nucleate MTs have been described [19], while in monkey cells in which the centrosome has been removed by microsurgery aMTOCs containing Pericentrin could be observed integrating into the mitotic spindle [34]. Furthermore, imaging of spindle formation in pig kidney cells showed that even in the presence of centrosomes peripheral, non-centrosomal MT clusters form and are utilized in spindle formation [35]. Interestingly, the ability to form aMTOCs appears to be upregulated in several cancer cell lines that still contain centrosomes; these aMTOCs lead to the formation of multiple spindle poles that need to be clustered into a bipolar spindle [36].

It is unclear, however, how aMTOCs are formed in somatic cells in the absence of centrioles. Moreover, although aMTOCs appear to contribute to spindle assembly in at least some systems [4,35,36] the significance of aMTOC mediated MT generation in spindle formation in somatic cells is still largely uncharacterized. In order to shed light on these open questions, we decided to study aMTOC formation and function in somatic *Drosophila* cells in vivo. We first set out to elucidate the pathway of aMTOC assembly. We found that aMTOCs consistently form in ~50–60% of mitotic fly somatic brain cells that lack centrioles, and that aMTOC assembly depends on the same proteins that are required to drive mitotic PCM assembly around the centrioles: Asl, Spd-2 and Cnn [37–45]. By identifying the proteins essential for aMTOC formation we then had means to specifically ablate formation of aMTOC formation in the absence of centrioles. Using these tools we performed a detailed genetic analysis to dissect the contribution of aMTOCs to mitotic spindle assembly in somatic *Drosophila* cells by comparing different acentriolar fly mutants, which either lack centrioles and aMTOCs or lack centrioles but form aMTOCs. Surprisingly, we find that aMTOCs do not detectably contribute to spindle assembly in the absence of centrosomes. In the absence of both centrosomes and MT nucleation from around the chromatin, however, aMTOCs significantly promote the assembly of monopolar spindles. Most importantly, we show for the first time that aMTOCs are essential for dynein-mediated acentriolar spindle pole focusing. On the basis of these observations we propose a revised model for acentriolar spindle organization by the molecular motors dynein and Ncd.

## Results

### *Drosophila* cells without centrioles can form aMTOCs in vivo that recruit several PCM components, nucleate microtubules and cluster at mitotic spindle poles

**Fig 1:**
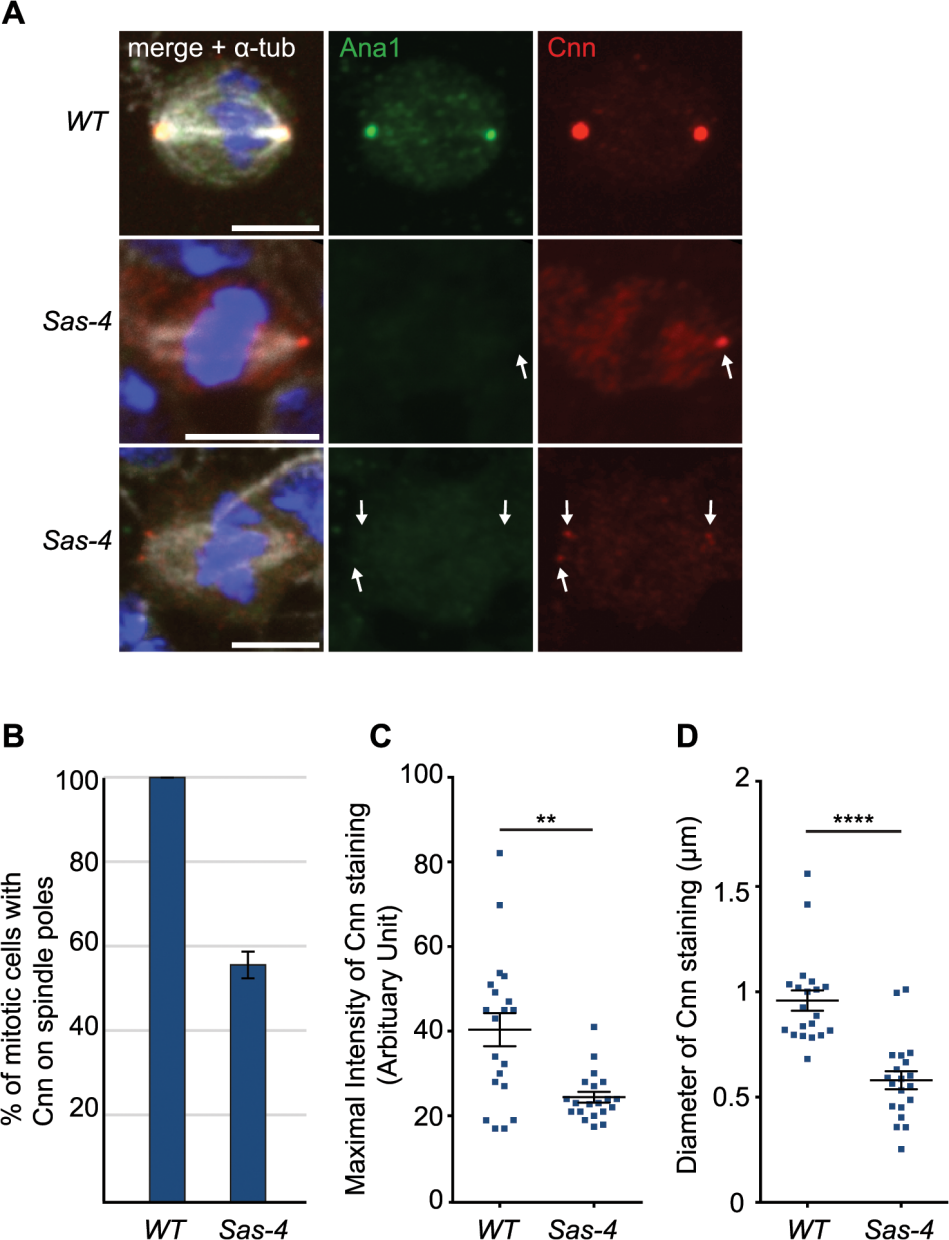
*Drosophila* cells without centrioles can form acentriolar microtubule organizing centers (aMTOCs). (A) *Drosophila* larval brain cells were stained with antibodies against Ana1 (green) to mark the centrioles, Cnn (red) to mark the PCM and α-tubulin (white) to mark the spindle. DNA is in blue. *Sas-4* mutants do not have centrioles as indicated by the lack of Ana1 on the spindle poles, but often have some Cnn detectable at either one (middle panel) or both (lower panel) of their mitotic spindle poles (arrows). *WT* and *Sas-4* cells were recorded using identical microscope settings, visualising the much fainter staining of Cnn on spindle poles in *Sas-4* mutant cells. Scale bars represent 5μm. (B) Quantification of Cnn recruitment to mitotic spindle poles in *WT* and *Sas-4* mutant cells (as judged by Cnn staining on at least one spindle pole) shows that Cnn staining on spindle poles can be detected in 100% ± 0 of *WT* cells and 55.93% ± 3.16 of *Sas-4* cells. A minimum of 120 cells from 4 brains were quantified per genotype. Data are expressed as mean±SEM with average per brain representing one data point. (C, D) Quantification of Cnn intensity (C) and the size of the Cnn foci (D) in *WT* or *Sas-4* mutant *Drosophila* brain cells stained with anti-Ana1 as centriole marker, anti-α-tubulin to visualise spindles and anti-Cnn. Cnn foci on 20 different spindle poles were analysed for each genotype. Data are expressed as mean±SEM with Cnn staining on each spindle pole representing one data point. P-values were calculated using a Mann-Whitney test.

It had previously been shown that γ-tubulin-containing aMTOCs can be found on spindle poles of acentriolar *Drosophila* cultured cell lines [33] and in *Drosophila* cultured cells in which centrioles have been depleted by knocking down the levels of the centriole duplication protein *Sas-4* by RNAi [32]. In cell lines derived from *Sas-4* mutants, however, aMTOCs could not be observed [46]. To test whether PCM proteins were detectable at acentriolar spindle poles in vivo we examined *Drosophila Sas-4* mutant larval brain cells. Electron microscopy studies have shown that these mutant cells lack detectable centrioles [16]. Staining for the centriolar protein Ana1 confirmed the lack of centrioles in *Sas-4* mutants (Fig 1A). Despite the absence of centrioles, however, we noticed localisation of the PCM protein Cnn at one or both spindle poles in ~56% of *Sas-4* mutant brain cells (Fig 1A and 1B). The staining of these Cnn foci was on average ~60% fainter than Cnn staining on centrosomes in *WT* cells (Fig 1C), and the acentriolar Cnn foci were on average ~60% smaller in diameter than centrosomes (Fig 1D). These observations support the previous conclusion that *Sas-4* mutant brain cells lack centrosomes [16], but suggest that about half of these cells have some detectable Cnn at least one spindle pole.

**Fig 2:**
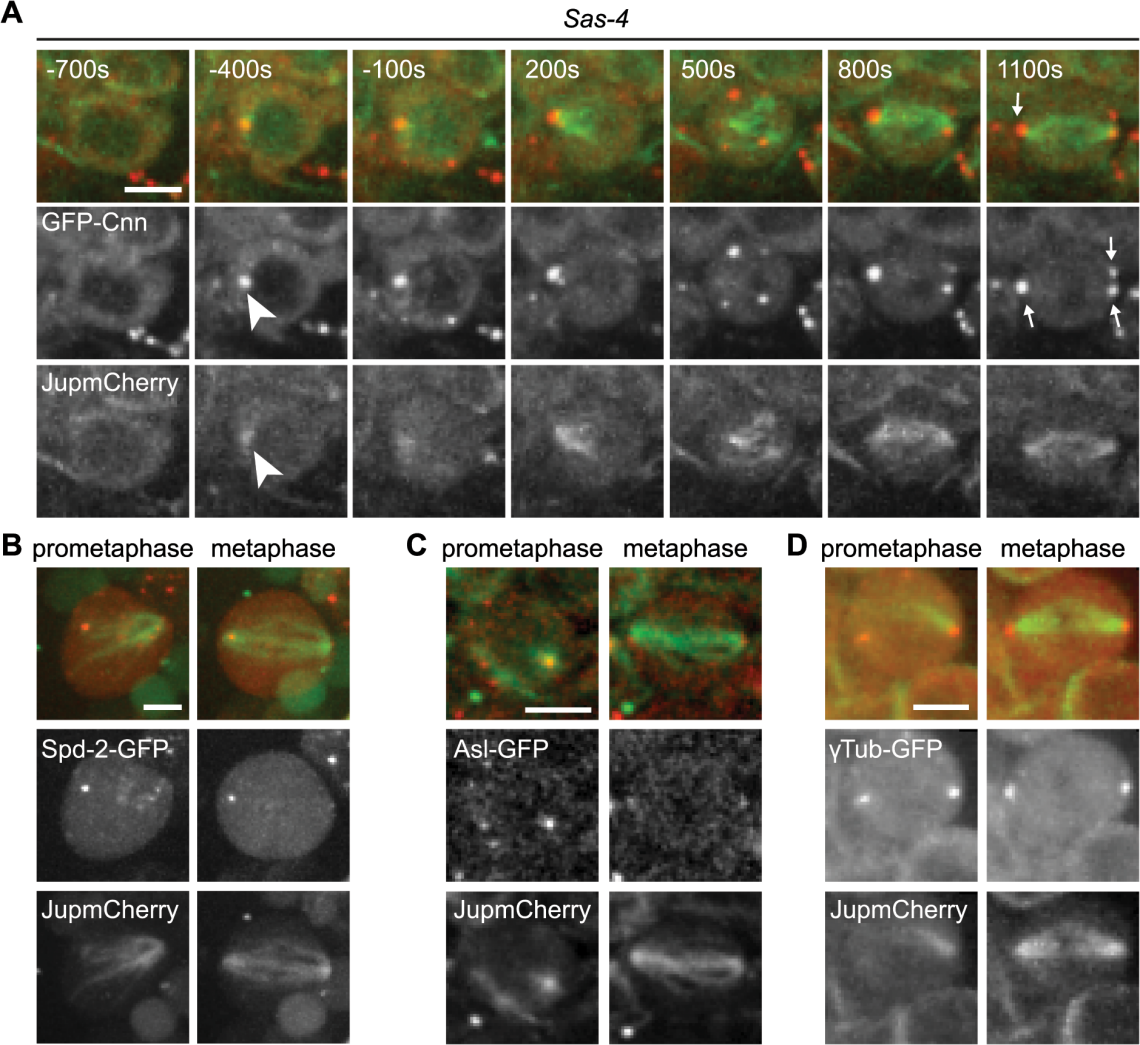
The formation of aMTOCs in living third instar larval brain cells. (A) Panels show images from time-lapse movie illustrating spindle formation in *Sas-4* mutant larval brain cells expressing GFP-Cnn (red) and Jupiter-mCherry (green). Time (secs) is indicated relative to nuclear envelope breakdown (NEBD). GFP-Cnn foci form in the cytoplasm and nucleate microtubules a few minutes prior to NEBD (arrowheads). These subsequently cluster at the spindle poles by metaphase (arrows). aMTOCs were present at metaphase in 54% of cells filmed (n = 55). aMTOCs are present in prometaphase and metaphase images from *Sas-*4 mutant brain cells expressing (B) Spd-2-GFP (red) and Jupiter-mCherry (green) (46%, n = 13), (C) Asl-GFP (red) and Jupiter-mCherry (green) (46%, n = 24), (D) γ-Tubulin-GFP (red) and Jupiter-mCherry (green) (54%, n = 59). All scale bars represent 5μm.

To analyse the mechanism of recruitment of Cnn to acentriolar spindle poles we analysed GFP-Cnn behaviour, together with Jupiter-mCherry as a MT marker, in living *Sas-4* mutant cells. As cells prepared to enter mitosis (judged by nuclear envelope breakdown—NEBD), a small number of GFP-Cnn dots began to appear in the cytoplasm of some cells, from which MTs then appeared to emanate (Fig 2A, arrowheads, S1 Movie). As cells entered mitosis, more Cnn dots of variable size and brightness became visible; these often associated with MTs and they tended to become clustered at the spindle poles as mitosis proceeded (Fig 2A, arrows). Importantly, a similar pattern was observed when we followed the behaviour of the centrosomal proteins Spd-2-GFP, γ-tubulin-GFP and Asl-GFP (although the levels of Asl-GFP in these dots was very low and no dots were detectable using anti-Asl antibodies in fixed cells—S1 Fig) (Fig 2B–2D). Based on the existing literature, we hereafter refer to these PCM foci as aMTOCs.

The PCM proteins PLP, Grip71WD, Msps, Aurora A and TACC all co-localised to some extent with the Cnn-containing aMTOCs in fixed *Sas-4* mutant cells, strongly suggesting that these structures contain many of the proteins normally concentrated at centrosomes during mitosis (S1 Fig). We conclude that, as in S2 cells depleted of *Sas-4* [32], aMTOCs can form during mitosis in *Drosophila* cells lacking centrioles in vivo. These aMTOCs appear to contain several known PCM proteins, and are formed prior to mitosis in the cytoplasm where they organize MTs that gradually become localized to the mitotic spindle poles.

### aMTOC formation is dependent on the known centrosomal PCM recruitment factors Asl, Spd-2 and Cnn

Next, we wanted to investigate the molecular pathway of aMTOC assembly. It has recently been shown that mitotic PCM recruitment in flies is largely dependent on just three proteins: the centriole duplication protein Asl, which is recruited to the outer region of the mother centriole, and the PCM scaffolding proteins Spd-2 and Cnn [38]. These are recruited to mother centrioles in an Asl-dependent manner, and assemble into a scaffold-structure that spreads outwards from the centrioles and recruits most other mitotic PCM components [37,38,47]. The centriole duplication protein Sas-4 has also been implicated in PCM recruitment [48,49], so we first tested which, if any, of the core centriole duplication proteins Sas-6, Ana2, Sas-4 or Asl are required for aMTOC assembly.

**Fig 3:**
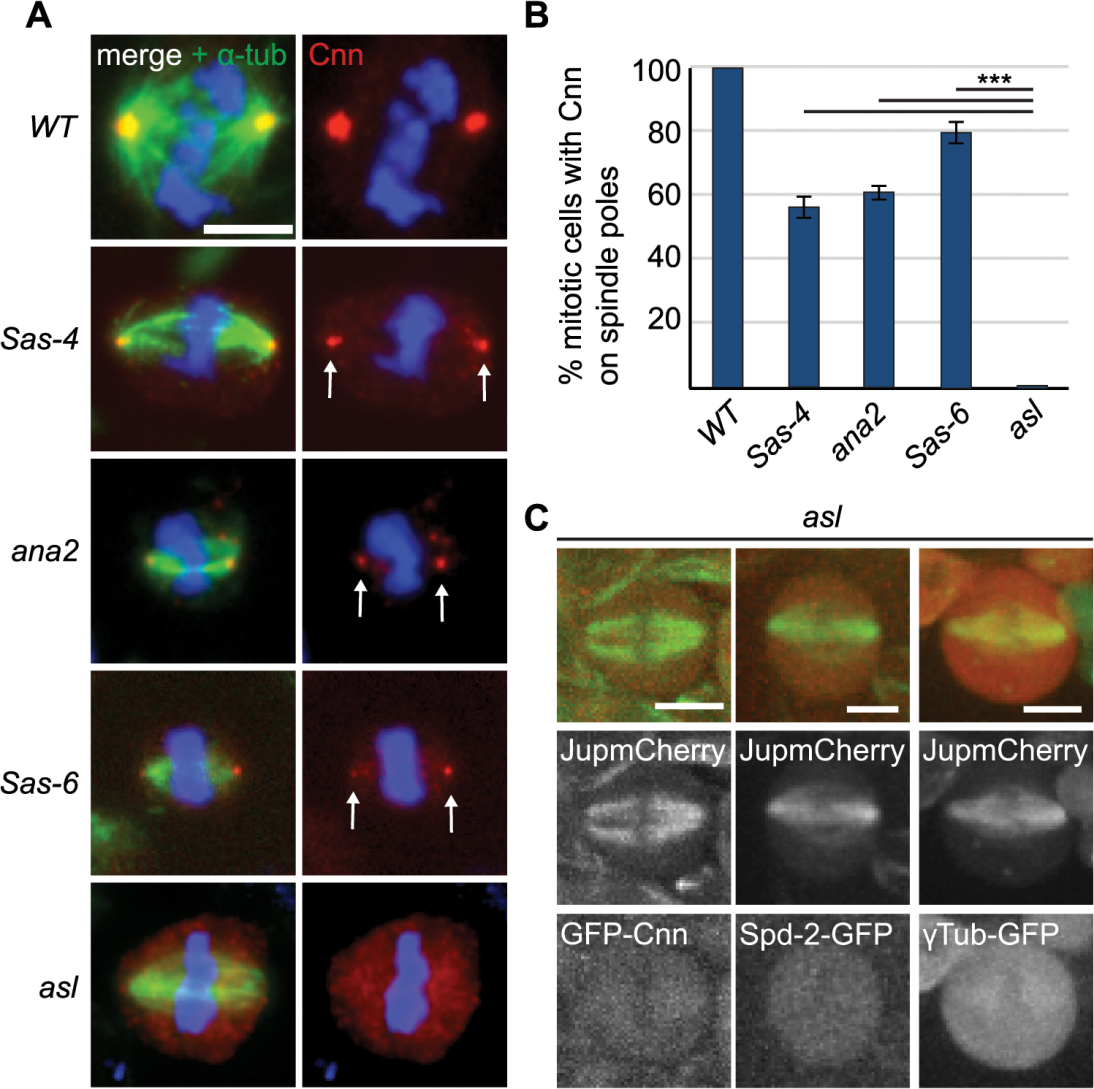
The PCM recruitment factor Asl is essential for aMTOC formation. (A) Larval brain cells of acentriolar *Drosophila* mutants were stained with antibodies against α-tubulin (green) and Cnn (red). DNA is in blue. aMTOCs are indicated by the arrows; note the absence of aMTOCs in *asl* mutants. (B) Quantification of aMTOC formation in mitotic brain cells (as judged by Cnn staining on at least one spindle pole) in acentriolar mutants shows that Cnn staining on spindle poles can be detected in 100% ± 0 of *WT* cells, 55.93% ± 3.16 of *Sas-4* cells, 60.46% ± 2.42 of *ana2* cells, 79.22% ± 3.87 of *Sas-6* cells and 0.68% ± 0.44 of *asl* cells. (A minimum of 120 cells from 4 brains were quantified per genotype). Data are expressed as mean±SEM with average per brain representing one data point. P-values were calculated using a Mann-Whitney test. (C) Spindle formation was followed in live *asl* mutant brain cells. No aMTOC formation can be observed in *asl* mutants throughout mitosis in stocks expressing Jupiter-mCherry (green) and either GFP-cnn (red) (n = 21), Spd-2-GFP (red) (n = 12) or γ-Tubulin-GFP (red) (n = 17). All scale bars represent 5μm.

We quantified Cnn staining on mitotic spindle poles in *Sas-6*, *ana2*, *Sas-4* and *asl* mutants, which all lack centrioles in third instar larval stages [16,50–52]. Interestingly, robust aMTOCs (identified by Cnn staining on at least one spindle pole) were present in ~50–60% of mitotic cells from *Sas-6*, *ana2* and *Sas-4* mutants, but were essentially undetectable in *asl* mutants (Fig 3A and 3B), and the same was true when we used other PCM proteins as aMTOC markers (S2 Fig). Moreover, Spd-2-GFP, GFP-Cnn and γ-tubulin-GFP did not detectably form aMTOCs in living *asl* mutant brain cells (Fig 3C). We conclude that Asl is essential for aMTOC formation in these cells.

**Fig 4:**
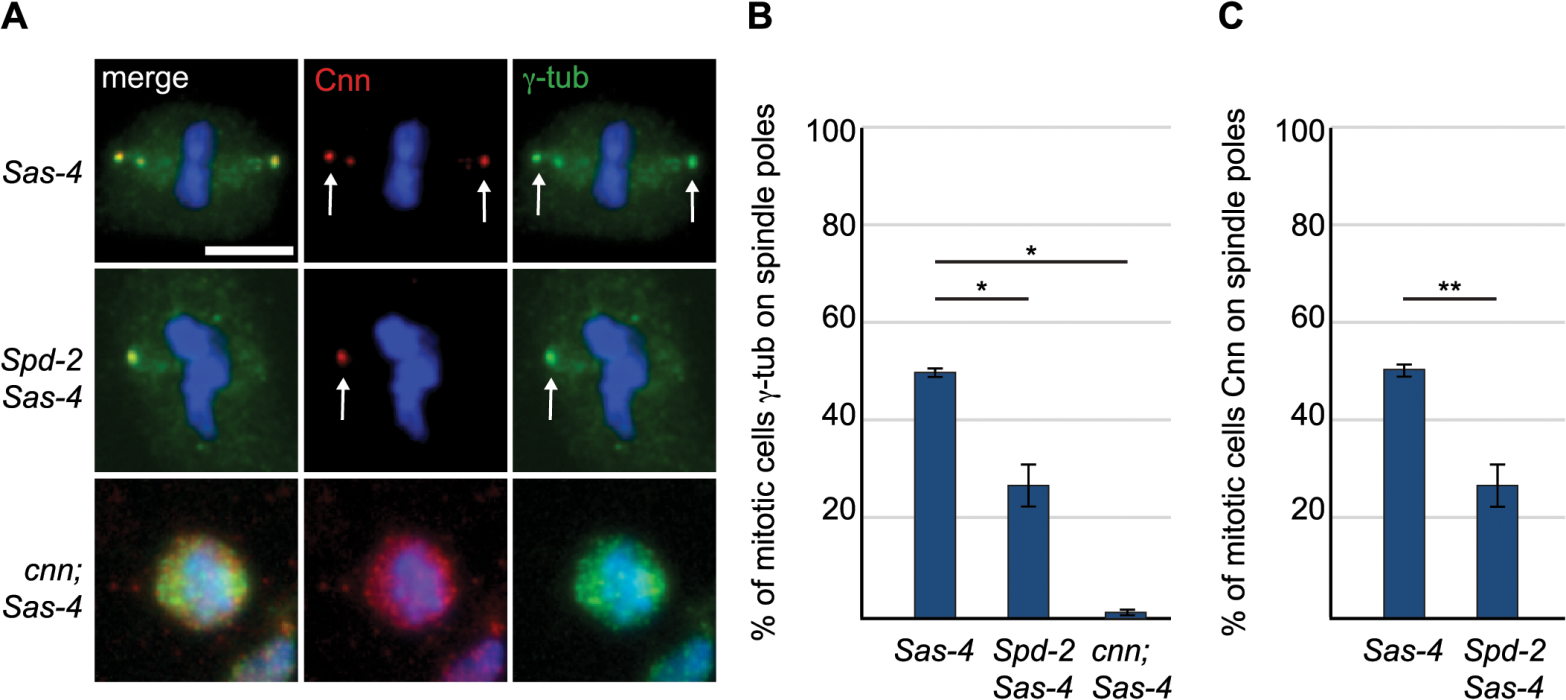
The PCM scaffolding proteins Spd-2 and Cnn are required for efficient aMTOC formation. (A) *Sas-4* mutant cells and *Spd-2 Sas-4* and *cnn; Sas-4* double mutant cells were stained with antibodies against Cnn (red) and γ-tubulin (green). DNA is in blue. Scale bar represents 5μm. The aMTOCs present in *Sas-4 and Spd-2 Sas-4* mutant cells are indicated by arrows. (B) Quantification of aMTOC formation in mitotic brain cells (as judged by γ-tubulin staining on at least one spindle pole) shows that γ-tubulin staining on spindle poles can be detected in 50.69% ± 0.83 of *Sas-4* mutant cells, 27% ± 4.33 of *Spd-2 Sas-4* double mutant cells and 1.23% ± 0.67 of *cnn; Sas-4* double mutant cells (a minimum of 80 cells from 3 brains were quantified per genotype). (C) Quantification of Cnn recruitment to mitotic spindle poles shows that Cnn staining on spindle poles can be detected in 50.69% ± 0.83 of *Sas-4* mutant cells and 27% ± 4.33 of *Spd-2 Sas-4* double mutant cells. (A minimum of 137 cells from 4 brains were quantified per genotype). All data are expressed as mean±SEM with average per brain representing one data point. P-values were calculated using a Mann-Whitney test.

We wondered whether Asl, by analogy to the centrosomal mitotic PCM recruitment pathway [38], was able to initiate PCM formation in the cytoplasm even in the absence of centrioles. This might happen by recruitment of Spd-2 and Cnn to cytoplasmic Asl, which could then provide a scaffold for PCM recruitment. We therefore examined PCM localisation on mitotic spindle poles in *cnn; Sas-4* and *Spd-2 Sas-4* double mutant strains, using the PCM protein γ-tubulin to assess the presence of aMTOCs. While ~51% of mitotic *Sas-4* mutant cells had detectable γ-tubulin foci on spindle poles, this was reduced to ~27% in *Spd-2 Sas-4* mutant cells and to only ~1% in *cnn; Sas-4* mutant cells (Fig 4A and 4B). Thus, Cnn appears to be essential for aMTOC formation, while Spd-2 has an important, but more minor role. Indeed, Spd-2 may function through its effect on Cnn recruitment [38] as Cnn was less efficiently localised at acentriolar spindle poles in the absence of Spd-2 (Fig 4C). Taken together these data suggest that aMTOC formation during mitosis is dependent on the same set of proteins that are essential for mitotic centrosome assembly.

### aMTOCs do not detectably increase the efficiency of spindle assembly in fly larval brain cells that lack centrosomes

These observations provided us with a strategy to genetically investigate the potential function of aMTOCs in acentriolar spindle assembly. *Sas-4* mutant cells lack centrioles, but have aMTOCs, while *asl* mutants lack both structures. Thus, by comparing the behaviour of *Sas-4* and *asl* mutant cells we can infer the contribution of aMTOCs to potentially any acentriolar biological process.

**Fig 5:**
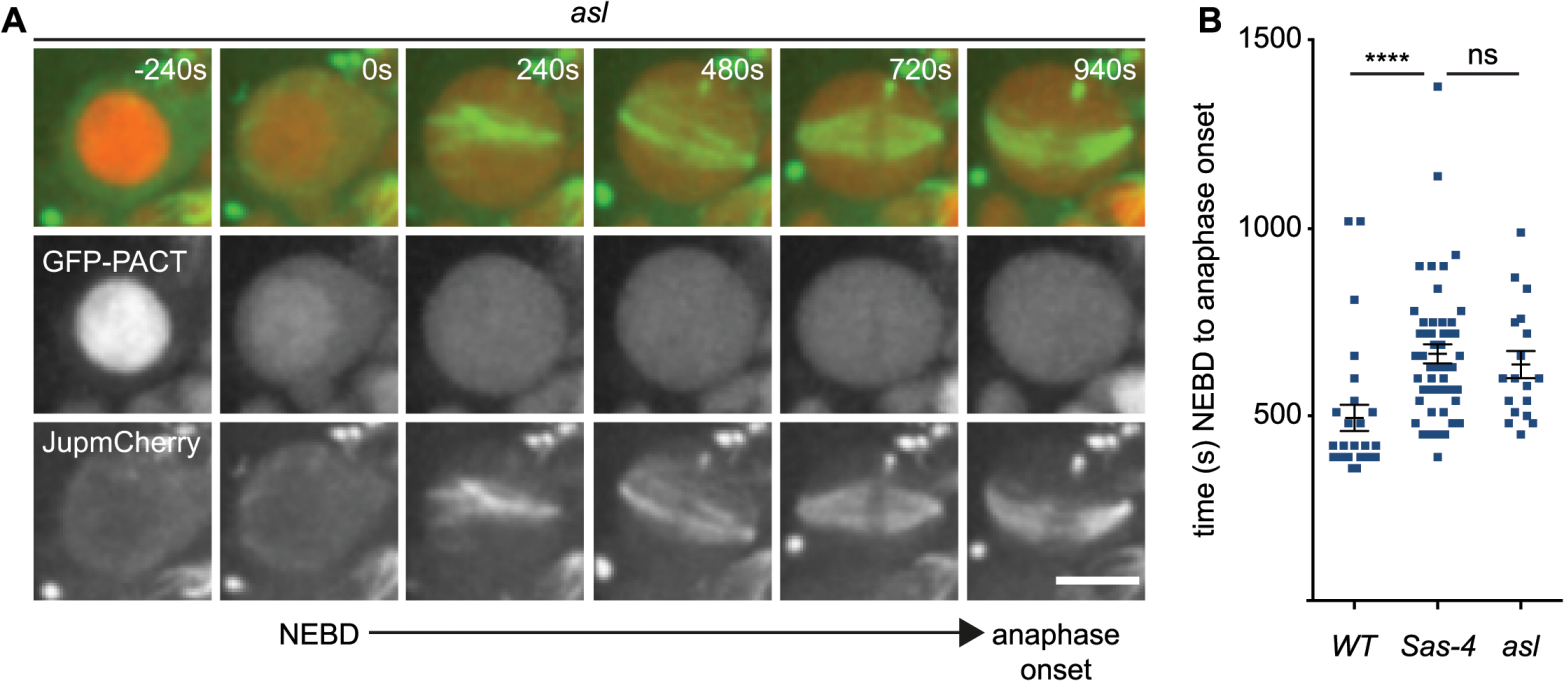
aMTOCs do not detectably contribute to spindle assembly in the absence of centrosomes. (A) *WT*, *asl* and *Sas-4* neuroblasts expressing GFP-PACT to mark centrioles and Jupiter-mCherry to visualise spindle formation were filmed to compare spindle assembly dynamics (*asl* is shown here as an example, time (secs) is relative to NEBD). Note how the GFP-PACT signal present in the nucleus at the first timepoint (-240s) is lost at NEBD. Scale bar represent 5μm. (B) Time between NEBD and anaphase onset was measured for *WT* (n = 27; 494s ± 35s), *Sas-4* (n = 50; 665s ± 25.6s) and *asl* (n = 18; 637s ± 36s) cells. Spindle assembly takes significantly longer in the mutant cells, but does not vary significantly between *Sas-4* and *asl* mutants indicating that aMTOCs do not increase the efficiency of spindle assembly. Data are expressed as mean±SEM. P-values were calculated using a Mann-Whitney test.

We first assessed the contribution of aMTOCs to acentriolar mitotic spindle assembly using time-lapse microscopy in mutant neuroblasts expressing the MT marker Jupiter-mCherry and the centriole marker GFP-PACT [53]. The latter protein also concentrates in nuclei during interphase, allowing us to precisely determine the time of NEBD [54] (Fig 5A and S2 Movie). As previously observed both *Sas-4* and *asl* mutant cells were able to form acentriolar spindles [16,40]. On average, both *asl* and *Sas-4* mutant neuroblasts were significantly delayed in spindle assembly compared to *WT*, confirming previous data in *Drosophila* and DT40 chicken cells lacking centrioles [16,19]. Surprisingly, however, there was no detectable difference in the timing of NEBD to anaphase onset between *asl* and *Sas-4* mutants (Fig 5B), suggesting that aMTOCs do not detectably contribute to the efficiency of spindle assembly in these cells.

### aMTOCs increase the efficiency of monopolar spindle formation in acentriolar cells that also lack misato

In the absence of centrosomes, the bulk of spindle MTs are nucleated by the chromatin-mediated pathway [55]. We wondered, therefore, whether the contribution to MT nucleation by aMTOCs could be masked by MT nucleation from around the chromatin. In flies, a mutation in *misato (mst)* inhibits regrowth of MTs from around the chromatin; *misato (mst)* mutants are larval lethal, and mutant mitotic cells fail in bipolar spindle assembly and usually only organize monopolar spindles of low MT density [56]. Cells that lack both Mst and centrioles can grow acentriolar MT arrays that appear to be nucleated from cytoplasmic foci, but the nature of these foci is unclear [56].

**Fig 6:**
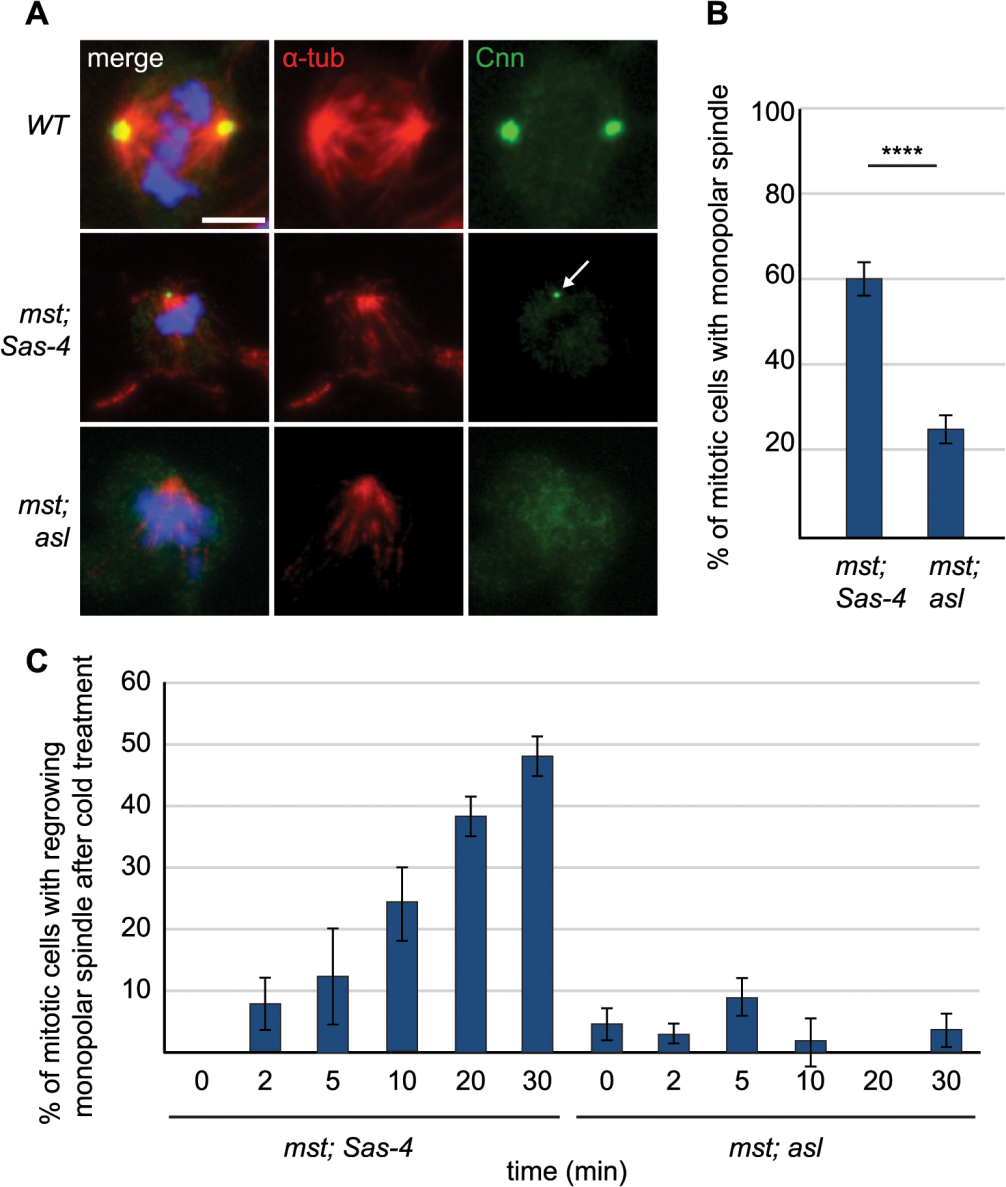
aMTOCs aid formation of monopolar spindles in acentriolar cells that also lack misato. (A) Larval brain cells of *WT*, *mst; Sas-4* and *mst; asl* double mutants were stained with antibodies against α-tubulin (red) and Cnn (green), DNA is in blue. *mst; Sas-4* cells have aMTOCs (arrow) on the poles of monopolar spindles. Scale bar represents 5μm. (B) Quantification of monopolar spindles in *mst; Sas-4* and *mst; asl* cells (a minimum of 263 cells in 8 brains were quantified per genotype). In *mst; Sas-4* mutants 59.32% ± 3.81 of cells form monopolar spindles while in *mst; asl* only 25.15% ± 3.34 of cells form monopolar spindles (the remaining cells do not exhibit any monopolar or bipolar spindles). (C) Quantification of regrowth of monopolar spindles after cold-induced depolymerisation at different time points after release from cold treatment. In *mst; Sas-4* brains 0% ± 0 of cells formed monopolar spindles at 0 minutes, 8% ± 4.25 at 2 minutes, 12.5% ± 7.89 at 5 minutes, 24.24% ± 6.1 at 10 minutes, 38.27% ± 3.24 at 20 minutes and 47.96% ± 3.09 at 30 minutes. In *mst; asl* brains 4.7% ± 2.62 of cells formed monopolar spindles at 0 minutes, 3.03% ± 1.55 at 2 minutes, 8.93% ± 2.9 at 5 minutes, 2% ± 3.93 at 10 minutes, 0% ± 0 at 20 minutes and 3.74% ± 2.74 at 30 minutes (a minimum of 43 cells from 4 brains were quantified for each timepoint). All data are expressed as mean±SEM with average per brain representing one data point. P-values were calculated using a Mann-Whitney test.

To test whether aMTOCs are formed in the absence of centrioles and the chromatin-mediated spindle assembly pathway we stained *mst; Sas-4* and *mst*; *asl* double mutant cells with antibodies against α-tubulin and Cnn. Cnn stained aMTOCs on the poles of monopolar spindles in *mst; Sas-4* double mutant cells, but not in *mst*; *asl* double mutant cells (Fig 6A). To test whether aMTOCs aid in mitotic array formation in the absence of both centrioles and the chromosome-mediated spindle assembly pathway we quantified mitotic cells with monopolar spindles. We found that approximately 60% of mitotic *mst; Sas-4* cells contained monopolar spindles, while only ~25% of *mst*; *asl* double mutant cells contained monopolar spindles of MTs (Fig 6B). We conclude that aMTOCs help to establish and/or maintain the monopolar spindles formed in cells lacking both the centrosomal and chromatin pathways of spindle assembly.

To test whether aMTOCs nucleate these monopolar MT arrays, or whether they are simply recruited to, and then help to stabilize them, we performed a MT-regrowth assay. In mitotic *mst; Sas-4* cells the MTs often grew back from one or several foci in the cytoplasm [56] and we found that these foci were often marked with Cnn (S3A Fig). After ~10 minutes of re-growth, the MTs had all usually coalesced into one acentriolar monopolar array that often had one Cnn-marked aMTOC at the pole (S3A Fig). Quantification of the formation of MT arrays over time showed that in mitotic *mst; Sas-4* cells the number of cells with monopolar MT arrays grew steadily over time until, after 30 minutes, ~50% of cells contained monopolar MT arrays again (Fig 6C). Remarkably, in *mst; asl* cells almost no regrowth of MT arrays was observed, and less than 5% of cells exhibited any MT arrays even after 30 minutes (Figs 6C and S3B). Thus, aMTOCs can nucleate MTs in mitotic cells, and they are an important source of MT generation in mitotic cells lacking centrosomal and chromatin MT nucleation.

### aMTOCs and dynein provide a mechanism for acentriolar spindle pole focusing in the absence of Ncd

In addition to providing a site for MT nucleation during mitosis, centrosomes are also involved in spindle pole focusing. In many systems spindle pole focusing in the absence of centrosomes has been shown to rely on the MT crosslinking kinesin Ncd [22–25] and the dynein complex [25–28]. The mechanism by which dynein contributes to acentriolar pole focusing is unclear, as its primary function in pole focusing in unperturbed cells is to transport K fibers towards centrosomes [9].

**Fig 7:**
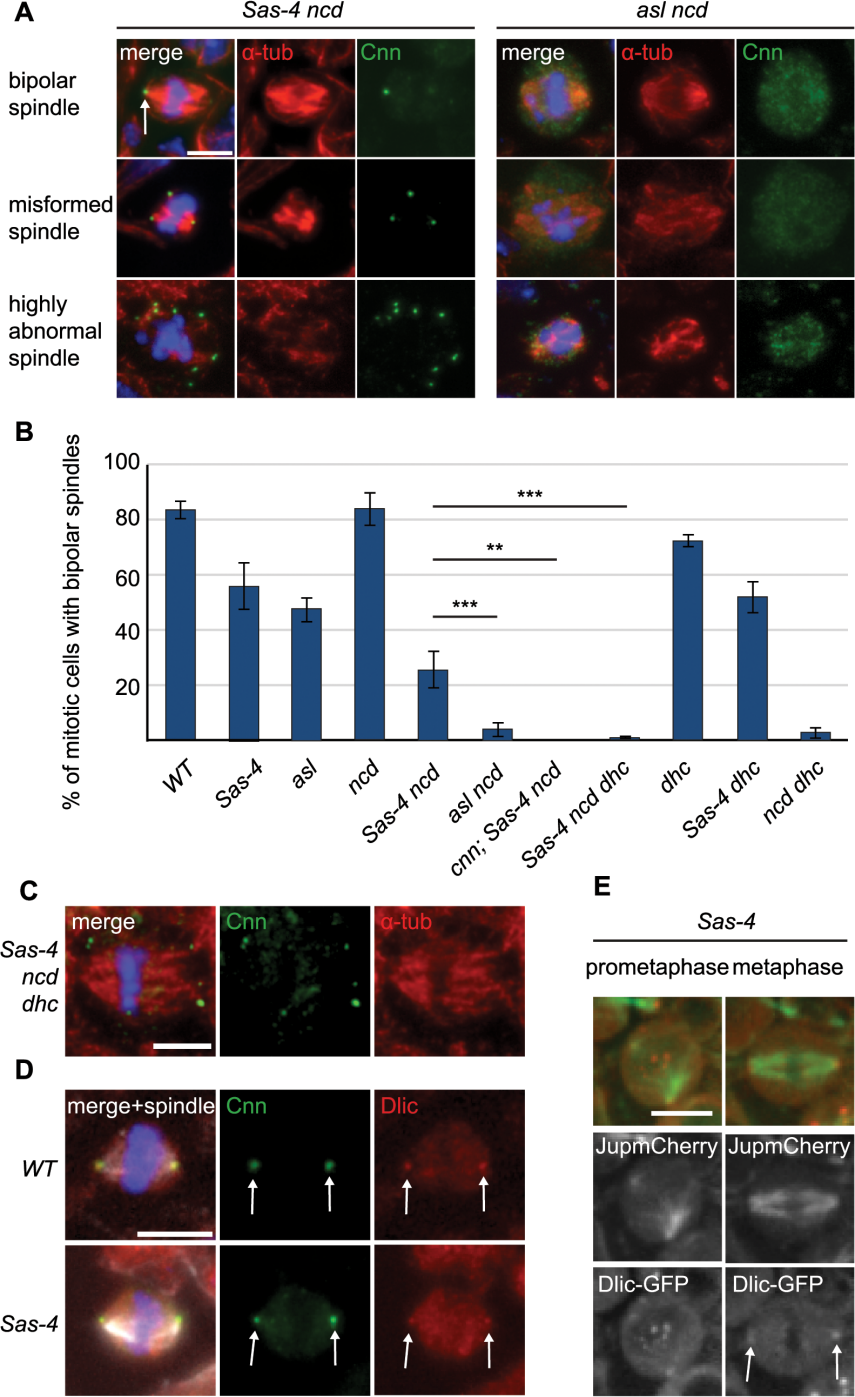
aMTOCs are essential for acentriolar spindle pole focusing by dynein. (A) Larval brain cells of *Sas-4 ncd* and *asl ncd* were stained with antibodies against Cnn (green) and α-tubulin (red). DNA is in blue. The different spindle phenotypes observed are illustrated; aMTOCs are present in *Sas-4 ncd* mutant cells (arrow). (B) Quantification of bipolar spindle formation ability in mitotic cells of different mutants (a minimum of 70 cells from 5 brains were quantified per genotype). 82.74% ± 2.94 of *WT* cells, 55.24% ± 8.11 of *Sas-4* cells, 47.08% ± 4.5 of *asl* cells, 83.65% ± 5.58 of *ncd* cells, 25.5% ± 6.47 of *Sas-4 ncd* cells, 4% ± 2.36 of *asl ncd* cells, 0% ± 0 of *cnn; Sas-4 ncd* cells, 0.73% ± 0.55 of *Sas-4 ncd dhc* cells, 71.95% ± 2.39 of *dhc* cells, 51.71% ± 5.67 of *Sas-4 dhc* cells and 2.72% ± 1.8 of *ncd dhc* cells formed bipolar spindles. All data are expressed as mean±SEM with average per brain representing one data point. P-values were calculated using a Mann-Whitney test. aMTOCs cooperating with dynein ameliorate the spindle formation defects observed in the absence of centrioles and Ncd (for a more detailed explanation see main text). Note that *Sas-4* and *asl* have fewer mitotic cells with formed spindles as spindle formation takes longer in the absence of centrioles (Fig 4). (C) *Sas-4 ncd dhc* triple mutant cells stained with antibodies against Cnn (green) and α-tubulin (red); these cells have a few misformed spindles with splayed spindle poles. (D) *WT* and *Sas-4* mutant cells stained with antibodies against Cnn (green) and Dynein light intermediate chain (red); Dynein localises to centrosomal MTOCs and aMTOCs (arrows). (E) Prometaphase and metaphase images from *Sas*-*4* mutant brain cells expressing Dlic-GFP (red, localising to aMTOCs as marked by the arrows) and Jupiter-mCherry (green) (The punctate signals in the middle region of the cell in prometaphase are likely to be localisation of Dlic-GFP to kinetochores). All scale bars represent 5μm.

In *Drosophila* Ncd is nonessential, but it is crucial for acentriolar meiotic spindle assembly in oocytes [22]. To test if Ncd is essential for acentriolar spindle formation in *Drosophila* somatic cells we generated *Sas-4 ncd* double mutants. Surprisingly, these cells were often still capable of forming bipolar mitotic spindles, although many cells exhibited defective spindle phenotypes, ranging from poorly focused and misformed to highly abnormal spindles (Fig 7A, left panels). Quantification of spindle assembly showed that these cells had a reduced ability to form bipolar spindles compared to *Sas-4* or *ncd* mutants alone, but were still able to form a spindle in ~26% of all mitotic cells (Fig 7B). As we often observed aMTOCs at the poles of the *Sas-4 ncd* cells that formed spindles (Fig 7A, arrow), we wondered whether these structures contributed to spindle formation in the absence of Ncd and centrioles. We therefore generated *asl ncd* double mutants to assess spindle formation in the absence of Ncd, centrioles and aMTOCs (Fig 7A, right panels). Quantification of the different spindle phenotypes showed that, strikingly, only 4% of mitotic *asl ncd* cells had formed a bipolar mitotic spindle, suggesting that aMTOCs indeed can ameliorate the severe spindle focusing defects observed in the absence of centrioles and Ncd. To confirm that it was the presence of aMTOCs, and not another potential intrinsic difference between *Sas-4* and *asl* mutants, that enhanced spindle focusing in the absence of Ncd we assessed spindle formation in *cnn; Sas-4 ncd* triple mutants. In addition to lacking centrioles these cells also cannot form aMTOCs, as Cnn is required for aMTOC formation (Fig 4A and 4B). We could not observe any bipolar mitotic spindles in *cnn; Sas-4 ncd* triple mutant cells, confirming our hypothesis that aMTOCs likely aid in spindle pole focusing in the absence of centrioles and Ncd (Fig 7B).

How do aMTOCs aid spindle formation in the absence of centrioles and Ncd? We hypothesised that dynein might transport K fibers along the MTs nucleated by aMTOCs to help focus spindle poles. If true, then cells which lack centrioles and Ncd but have aMTOCs would not form bipolar spindles if dynein was also lacking. To test this possibility we generated *Sas-4 ncd dhc (dynein heavy chain)* triple mutants and assessed spindle formation in these cells. Intriguingly, almost none of these cells showed bipolar mitotic spindles (~1%, Fig 7B) and the few spindles we observed invariably exhibited unfocussed poles (Fig 7C). To rule out that this phenotype could originate from another, aMTOC independent, role of dynein we assessed spindle formation in *dhc* single mutants and *Sas-4 dhc* double mutants. Both of these had relatively normal spindle morphology, suggesting that dynein in conjunction with aMTOCs is required to ameliorate spindle formation in the absence of Ncd (Fig 7B).

These data suggest that dynein function in spindle focusing might be to transport kinetochore MTs towards MTOCs—either centriolar or acentriolar. If so, cells which have aMTOCs but lack Ncd and dynein should have a very similar phenotype to cells which have centrosomes but lack Ncd and dynein: in both cases K fibers could not be transported towards (either acentriolar or centriolar) MTOCs on the spindle poles. To test this hypothesis, we generated *ncd dhc* double mutants (which have centrosomes but lack Ncd and dynein). Only ~3% of these mitotic cells formed a bipolar spindle, which was very similar to the situation in *Sas-4 ncd dhc* triple mutants (which have aMTOCs but lack Ncd and dynein) (Fig 7B). We conclude that dynein is able to focus acentriolar mitotic spindles in an analogous fashion to spindle focusing in centrosomal cells, namely by transporting K fibers towards aMTOCs. In support of this hypothesis, dynein clearly localised to centrosomes (as previously observed [57]) and also aMTOCs on mitotic spindle poles in fixed (Fig 7D) and live larval brain cells (Fig 7E and S3 Movie), but failed to localise in *asl* mutants (S4 Fig).

### Acentriolar spindle pole focusing by dynein and aMTOCs can ameliorate proliferation defects observed in the absence of centrioles and Ncd

**Fig 8:**
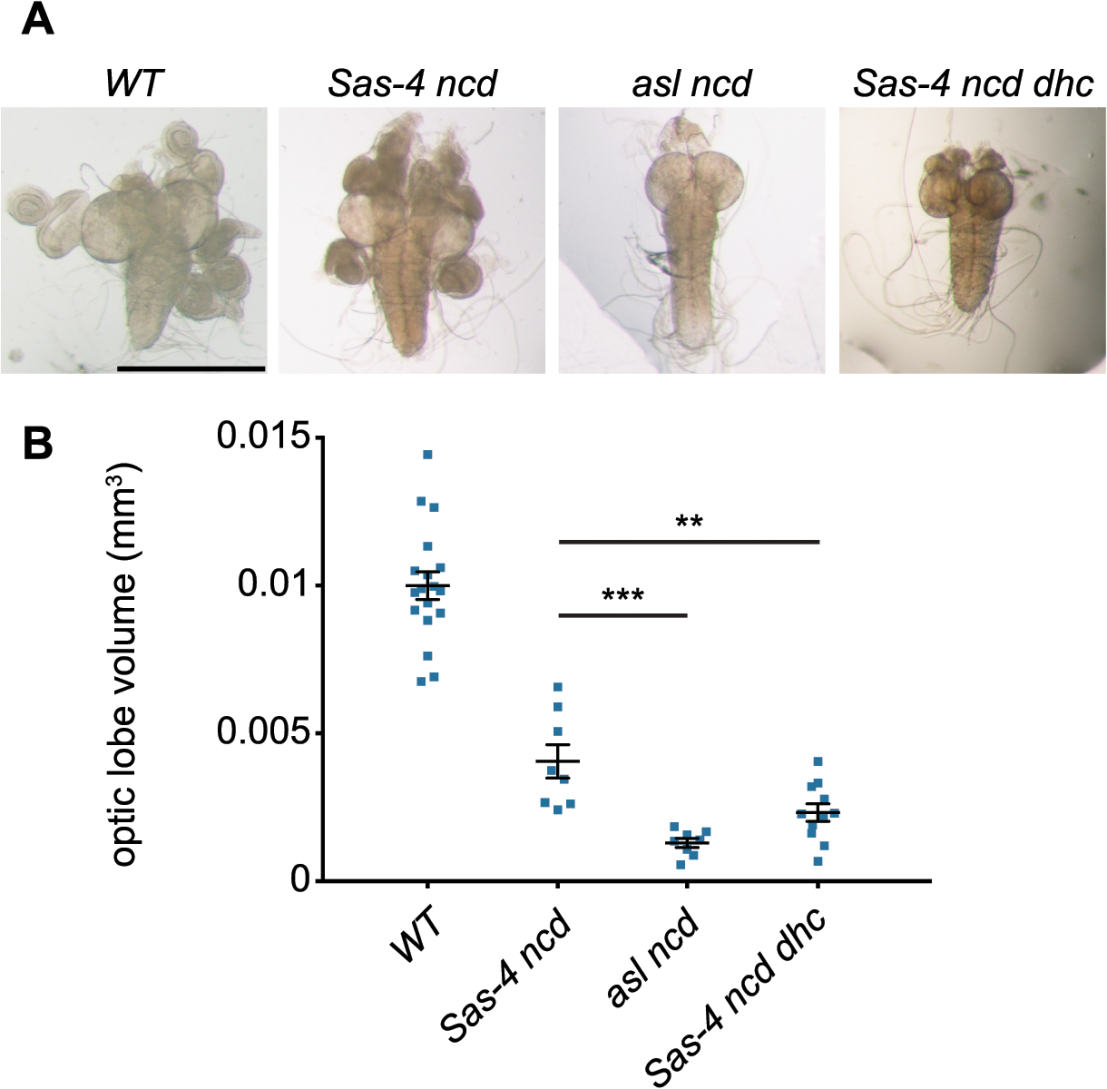
Acentriolar spindle pole focusing by dynein can ameliorate proliferation defects observed in the absence of centrioles and Ncd. (A) Panels show third instar larval brains from *WT* and different mutant strains. Note how *asl ncd* and *Sas-4 ncd dhc* brain lobes are much smaller than *WT* or *Sas-4 ncd* brains and lack fully developed imaginal discs. (B) Quantification of third instar larval brain size in *WT*, *Sas-4 ncd*, *asl ncd* and *Sas-4 ncd dhc* 3rd instar wandering larvae. Brain size was assessed by measuring brain lobe circumference and calculating brain lobe volume under the assumption that the lobes were spherical. Each data point represents the averaged brain lobe volume of each two lobes of a brain (a minimum of 8 brains were analysed per genotype). A Mann-Whitney test shows that brains that lack aMTOCs and Ncd (*asl ncd*) or have aMTOCs but lack dynein and Ncd (*Sas-4 ncd dhc*) brains are both significantly smaller than *Sas-4 ncd* brains (that lack Ncd but have aMTOCs and dynein) suggesting that acentriolar spindle focusing by aMTOCs and dynein can ameliorate the proliferation defects observed in the absence of Ncd and centrioles.

Finally, we wanted to assess whether the ability of dynein and aMTOCs to promote spindle formation in acentriolar cells might be sufficiently robust to allow these acentriolar cells to proliferate even in the absence of Ncd. We compared acentriolar *Sas-4 ncd* tissue (which lacks Ncd, but has aMTOCs) with *asl ncd* tissue (which lacks both Ncd and aMTOCs). We noticed that while *Sas-4 ncd* brains were slightly smaller than *WT* brains they still looked relatively normal and had imaginal discs attached to them (Fig 8A and 8B). In comparison *asl ncd* brains and imaginal discs were much smaller than normal (Fig 8A and 8B), indicating a much stronger defect in cell proliferation [58]. This suggested that the severe proliferation defect in *asl ncd* larvae was rescued by aMTOC and dynein-mediated spindle focusing in *Sas-4 ncd* larvae. In support of this interpretation, *Sas-4 ncd dhc* larvae (which have aMTOCs but lack dynein) also had very small brains and discs (Fig 8A and 8B). We conclude that aMTOCs and dynein can cooperate to focus acentriolar spindle poles in cells lacking Ncd, and that this pathway substantially increases the efficiency of spindle assembly and thereby the ability of these cells to proliferate.

## Discussion

Here we confirm the presence of aMTOCs in acentriolar somatic cells and show that the pathway of aMTOC assembly in fly somatic brain cells is genetically very similar to the pathway of mitotic centrosome assembly. At centrosomes, Asl is present exclusively around the mother centriole [59–61], where it helps recruit Spd-2 and Cnn, which then assemble into a scaffold that spreads outwards around the mother centriole and which ultimately recruits the other mitotic PCM components [37–45]. Our data suggest that in ~50–60% of mitotic brain cells that lack centrioles, Asl can still recruit some Spd-2 and Cnn to form cytoplasmic scaffold structures that can then recruit other PCM components to form aMTOCs. Thus, while mother centrioles greatly increase the efficiency of mitotic PCM assembly, these three proteins can self-assemble into a mitotic scaffold structure even in their absence. The number and size of the aMTOCs formed, however, was very variable, suggesting that centrioles not only make mitotic PCM assembly more efficient, but they also serve to regulate the amount of PCM assembled and to ensure that only two MTOCs are normally formed during mitosis.

Structures similar to the aMTOCs described here have also been observed in other *Drosophila* tissues. In oocytes bundles of MTs were observed in the cytoplasm during acentriolar meiotic spindle formation [22]. No centrosomal markers such as γ-tubulin and CP60 have been observed clustering on meiotic spindle poles [22,62,63] and therefore it is unlikely that these MT bundles represent aMTOCs. In *Drosophila* embryos, injection of an antibody raised against Spd-2 lead to displacement of PCM from the centrosomes and thereby removed centrosomally nucleated MTs. In these embryos MT asters could be observed to form in the cytoplasm, which were then organised into mitotic spindles [4]. As centrioles are still present in these embryos it is unclear, whether these asters were nucleated from self-assembled aMTOCs as described here, or whether they are fragments of PCM originally nucleated around the mother centriole and then displaced from the centrosomes either through a lack of Cnn or by Spd-2 antibody injection [4]. Finally, a study has described the biochemical purification of cytoplasmic complexes containing Sas-4, Cnn, Asl and Plp (S-CAP complexes; [64]) from *Drosophila* embryonic extracts. Although S-CAP complexes have a composition similar to the aMTOCs described here, they require Sas-4 for their assembly [64]. The aMTOCs we describe here form in the absence of Sas-4, suggesting that they are unrelated.

Our identification of the factors required for aMTOC assembly in *Drosophila* allowed us to perform a detailed genetic analysis of the contribution of aMTOCs to different aspects of mitotic spindle assembly in acentriolar fly cells. Surprisingly, we find that aMTOCs do not detectably increase the rate of spindle assembly in somatic brain cells that lack centrioles. This suggests that, in contrast to meiotic spindle assembly in mouse oocytes [30], aMTOCs do not play a major role in generating spindle MTs in the absence of centrosomes, at least in this cell type.

Our data show, however, that aMTOCs can function as a major source of MT regrowth in cells that lack both centrosomal- and chromatin-mediated MT nucleation after the MT cytoskeleton has been depolymerised by cold treatment. Before cold treatment *mst; asl* mitotic cells (that lack the chromatin-, centrosome- and aMTOC-pathway of MT assembly) still had mitotic MT arrays, but these were significantly fewer than *mst; Sas-4* cells (that lack the chromatin- and centrosome-pathways, but still have aMTOCs). We speculate that the MT arrays in *mst; asl* mitotic cells arise from augmin-mediated MT nucleation from pre-existing MTs, which can no longer happen once all MTs have been removed by cold-treatment.

**Fig 9:**
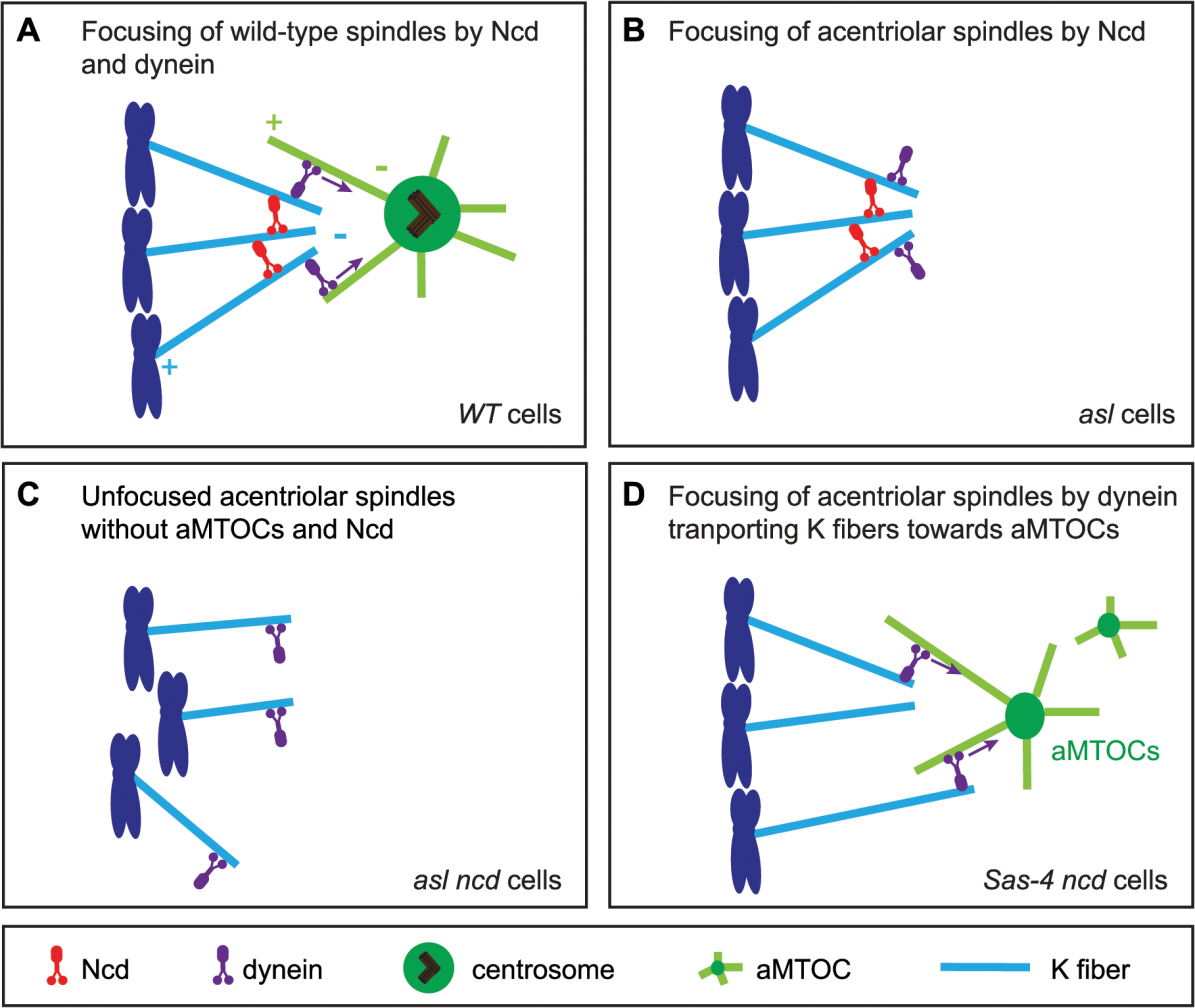
A model for acentriolar spindle pole focusing by the molecular motors dynein and Ncd. (A) In *Drosophila* cells with centrosomes K fibers (light blue) are thought to be predominantly crosslinked by the minus end-directed motor Ncd (red), while the minus-end directed motors dynein (purple) transports the K fibers along centrosomal MTs (light green) to the centrosome [9]. (B) In cells lacking centrosomes, Ncd can still focus acentriolar spindle poles by crosslinking the K fibers (as, for example, in an *asl* mutant cell). (C) Loss of Ncd in acentriolar cells which also lack aMTOCs leads to severe impairment of spindle focusing as dynein cannot crosslink MT minus ends (as, for example, in *asl ncd* mutant cells). (D) In acentriolar cells with aMTOCs (as, for example, in *Sas-4 ncd* mutant cells), dynein can transport K fibers towards aMTOCs and so allow some spindle pole focusing even in the absence of Ncd.

Interestingly, we find that aMTOCs do play an important part in the mechanism of acentriolar spindle focusing by dynein. Dynein usually transports MTs emanating from kinetochores along astral MTs to the centrosome [6,9]. Our observations suggest that dynein can also transport MTs towards aMTOCs. Based on our results we propose an updated model for acentriolar spindle pole focusing by the minus end directed motors Ncd and dynein (Fig 9). In *Drosophila* cells with centrosomes, K fibers become crosslinked by Ncd, while dynein transports K fibers along centrosomal MTs towards the centrosome [9] (Fig 9A). When centrosomes and aMTOCs are lost, Ncd becomes essential for focusing acentriolar spindle poles as it can crosslink K fibers independently of centrosomes (Fig 9B). Therefore loss of Ncd leads to severely unfocused poles in acentriolar cells, as dynein, in contrast to Ncd, does not have the ability to statically crosslink MT minus ends (Fig 9C). If aMTOCs are present, however, then dynein can partially compensate for the loss of Ncd by focusing spindle poles through the transport of K fibers towards aMTOCs (Fig 9D).

This last point is important, as previous studies that reported a role for dynein in acentriolar pole focusing did not describe the molecular mechanism by which dynein is able to do this [25–28]. We suspect that structures similar to aMTOCs must have been present in these earlier studies for dynein to fulfil its role. These studies used *Xenopus* egg or cell extracts for their analyses. Interestingly in Verde et al. 1991 [28] the observation of a MT crosslinking material that accumulated at MT minus ends is described, and electron microscopy showed that this material had electron-dense properties similar to PCM [28]. Our finding that dynein needs to cooperate with aMTOCs to focus acentriolar spindles is an important addition to our understanding of the mechanism of spindle pole coalescence by molecular motors.

## Material and Methods

### Fly strains

The following mutant alleles were used in this study: *Sas-4* ^*s2214*^ [16], *Sas-6* ^*c02901*^ [50,51], *asl* ^*B46*^ (this study), *Spd-2* ^*G20143*^ [43], *cnn* ^*f04547*^ [45], *cnn* ^*HK21*^ [44,65], *ana2* ^*169*^ [66], *mst* ^*LB20*^ [56], *dhc* ^*6-10*^ [67] and *ncd* ^*1*^ [68]. The following transgenic insertion lines were used: Asl::Asl-GFP [69], Ubq-GFP-Cnn [37], Ubq-Spd-2-GFP [43], ncd:: γ-tubulin37C-GFP [70], Ubq-Dlic-GFP (this study—a full length Dlic [dynein light intermediate chain—CG1938] cDNA was cloned into the Ubq-GFPNT Gateway vector [71]; Nina Peel, personal communication) and Jupiter-mCherry [72]. w^67^ was used as wild type control. An *asl* null allele was generated by inducing imprecise excision of the P-Element P{EPg}HP37249 located near the 5’UTR about 470bp upstream of the *asl* start codon. A 2.1kb deletion removing nearly 500bp upstream of the start codon and over half of the coding region was recovered (S4 Fig); the allele was named *asl* ^*B46*^. In contrast to previously published alleles of *asl* [69] this new allele *asl* ^*B46*^ produces no detectable N- or C-terminal Asl protein in western blotting or immunofluorescence experiments (S4 Fig). We used this new *asl* ^*B46*^ mutant as it represents a true null mutation with no residual part of Asl being expressed, however we observed that aMTOC formation is also ablated in previously published *asl* ^*mecD*^ allele [69] (Cnn staining on spindle poles can be detected in 0.55% ± 0.55 of *asl* ^*mecD*^ cells).

### Immunofluorescent staining and antibodies and microscopy

Fixation and stainings of third instar larval brains was performed as previously described [53]. The MT regrowth assay in larval brains was performed as described in [56]. The following primary antibodies were used at a 1:500 dilution: Rabbit anti-Dlic (this study—raised against amino acids 1–293 of the *Drosophila* Dlic (CG1938) coding sequence; Nina Peel, personal communication), rat anti-Asl [73], rabbit anti-Asl (N-terminal and C-terminal) [37], guinea pig anti-Cnn [45], rabbit anti-Spd-2 [43], mouse anti- γ-tubulin (GTU88, Sigma), mouse anti-actin (Sigma-Aldrich), rabbit anti-PLP [53], rabbit anti-TACC [74], rabbit anti-Grip71WD [75], rabbit anti-Msps [76], rabbit anti-Aurora A [77], mouse monoclonal anti-α-tubulin (DM1α, Sigma-Aldrich) and rabbit anti-Histone H3 Phospho S10 (Upstate Biotechnology). Alexa488 anti-guinea pig and anti-mouse and Alexa568 anti-rabbit as secondary antibodies were used at a 1:1000 dilution (Molecular Probes, Life Technologies). Fixed preparations were examined using either a Zeiss LSM780 confocal microscope using a 63x/1.40 NA objective, or on a Zeiss Axioskop 2 microscope (Carl Zeiss, Ltd) with a CoolSNAP HQ camera (Photometrics), using a 63x/1.25 NA objective (Carl Zeiss, Ltd). Images were processed with Fiji [78] and adjusted to use the full range of pixel intensities.

### Quantification of aMTOCs

Larval brains were stained with antibodies against α-tubulin to visualise spindles, Cnn to visualise aMTOCs and anti-Phospho-Histone H3 and were imaged on a Zeiss Axioskop 2 microscope. Only mitotic cells identified by Phospho-Histone H3 staining were scored, and all phenotypes were always quantified blindly, without knowing which genotype was being counted.

### Live imaging

Brains were dissected from third instar larvae in PBS and the attached imaginal discs were removed. The brain was transferred to a drop of PBS on a clean coverslip and covered with a slide, which semi-squashed the brain. The coverslip was sealed with a drop of Voltalef 10S oil (VWR) to stop evaporation of the PBS and brains were analysed using a 63x/1.4 NA lens on a Perkin Elmer ERS Spinning Disk confocal system mounted in an inverted microscope (Axiovert 200M; Carl Zeiss Ltd) with a charge-coupled device camera (Orca ER; Hamamatsu) with Ultraview ERS software (Perkin Elmer), or using a 60x/1.4 NA lens on an Andor Revolution XD Spinning Disk confocal system mounted on an inverted Nikon (TE-2000E) with an Electron Multiplying charge-coupled device camera (iXon, Andor) and IQ2 software (Andor).

### Analysis of larval brain size

Third instar larval brains were dissected and the brain lobe volume was measured as previously described [79].

## Supporting Information

S1 Fig Several PCM proteins localise to aMTOCs in *Sas-4* mutant cells. Fixed *Drosophila* brain cells from *WT* (left-panels) and *Sas-4* mutants (right-panels) were stained with antibodies against Cnn (red) and either γ-tubulin, PLP, Grip71WD, Msps, Aurora A, TACC or Asl (green). DNA is in blue. All of these PCM proteins colocalise with Cnn in mitotic *Sas-4* cells (indicated by arrows), except for Asl. Scale bar represents 5μm.
(EPS)

S2 Fig aMTOCs are not detectable in *asl* mutant cells. Fixed *Drosophila* brain cells from *asl* mutants were stained with antibodies against Cnn (red) and either γ-tubulin, PLP, Grip71WD, Msps, Aurora A or TACC (green). DNA is in blue. All of these PCM proteins were not detectable in aMTOCs in mitotic *asl* cells. Scale bar represents 5μm.
(EPS)

S3 Fig Microtubule regrowth assay in *mst; Sas-4* and *mst; asl* cells. (A) *mst; Sas-4* double mutant cells were fixed at different time points ranging from 0–10 minutes after reintroduction from ice to room temperature and stained with antibodies against α-tubulin (red) and Cnn (green). Mitotic MT arrays that had been depolymerized on ice start to regrow from often several Cnn marked aMTOCs in the cytoplasm. Small MT foci cluster together until most mitotic cells have one monopolar array. (B) Representative images of *mst; asl* double mutant cells 10 minutes after reintroduction to room temperature. Note the lack of monopolar spindles follow 10 minutes regrowth in *mst; asl* double mutant cells. Scale bar represents 5μm.
(EPS)

S4 Fig Dynein is not detectable at the poles of the mitotic spindle in *asl* mutants. (A) *asl* mutant cells stained with antibodies against Cnn (green) and dynein light intermediate chain (red). (B) Prometaphase and metaphase images from *asl* mutant brain cells expressing Dlic-GFP (red) and Jupiter-mCherry (green) (The punctate signals in the middle region of the cell in prometaphase are likely to be localisation of Dlic-GFP to kinetochores). All scale bars represent 5μm.
(EPS)

S5 Fig Characterization of the *asl* null allele *asl* ^*B46*^. (A) Schematic illustration of the *asl* genomic region with the sequence deleted in *asl* ^*B46*^ mutants indicated. (B) *asl* ^*B46*^ mutants eclose as morphologically normal adults but are severely uncoordinated. Expression of Asl-GFP rescues the uncoordinated phenotype. (C) Antibody staining of fixed WT; *asl* ^*B46*^ and *Asl-GFP*, *asl* ^*B46*^ mutant larval brain cells with antibodies recognising Asl, γ-tubulin and Phospho-Histone H3. In *asl* ^*B46*^ mutant mitotic cells no Asl protein and no centrioles can be detected. Asl-GFP rescues the loss of centrioles in *asl* ^*B46*^ mutants. (D) Western blot of *WT*, *asl* ^*mecD*^ mutants [69,75] and *asl* ^*B46*^ mutants (this study) with anti-Asl antibody recognizing the N-terminal end of Asl and the C-terminal end. In *asl* ^*mecD*^ a faint band of a small Asl fragment is still detected by the N-terminal antibody. No residual band is detected in *asl* ^*B46*^ mutants.
(EPS)

S1 Movie The formation of aMTOCs in living third instar larval brain cells. Timelapse movie of spindle formation in *Sas-4* mutant larval brain cells expressing GFP-Cnn (red) and Jupiter-mCherry (green). Movie shows both merged and separate channels. Time (secs) indicates time relative to NEBD.
(AVI)

S2 Movie Spindle formation in cells lacking centrosomes and aMTOCs. *asl* mutant neuroblasts expressing GFP-PACT (red) to mark centrioles and Jupiter-mCherry (green) to visualise spindle formation were filmed to assess spindle assembly dynamics in cells lacking both centrosomes and aMTOCs. Time (secs) indicates time relative to NEBD). (assessed as the time when nuclear GFP-PACT fluorescence levels were comparable to cytoplasmic GFP-PACT levels).
(AVI)

S3 Movie Dynein complex labels aMTOCs during acentriolar spindle formation. *Sas-4* mutant neuroblast expressing Dlic-GFP (red) and Jupiter-mCherry (green). Movie shows both merged and separate channels. (The punctate signals in the middle region of the cell following NEBD are likely to be localisation of Dlic-GFP to kinetochores). Time (secs) indicates time relative to NEBD.
(AVI)
